# Temperature Exerts Control of *Bacillus cereus* Emetic Toxin Production on Post-transcriptional Levels

**DOI:** 10.3389/fmicb.2016.01640

**Published:** 2016-10-25

**Authors:** Markus Kranzler, Katharina Stollewerk, Katia Rouzeau-Szynalski, Laurence Blayo, Michael Sulyok, Monika Ehling-Schulz

**Affiliations:** ^1^Functional Microbiology, Institute of Microbiology, Department of Pathobiology, University of Veterinary Medicine ViennaVienna, Austria; ^2^Food Safety Microbiology, Nestec Ltd, Nestlé Research CenterLausanne, Switzerland; ^3^Center for Analytical Chemistry, Department of Agrobiotechnology, IFA Tulln, University of Natural Resources and Life Sciences Vienna (BOKU)Vienna, Austria

**Keywords:** *Bacillus cereus*, cereulide, isocereulide, temperature, food safety

## Abstract

In recent years, the emetic toxin cereulide, produced by *Bacillus cereus*, has gained high relevance in food production and food safety. Cereulide is synthesized non-ribosomal by the multi-enzyme complex Ces-NRPS, which is encoded on a megaplasmid that shares its backbone with the *Bacillus anthracis* pX01 toxin plasmid. Due to its resistance against heat, proteolysis and extreme pH conditions, the formation of this highly potent depsipeptide toxin is of serious concern in food processing procedures including slow cooling procedures and/or storage of intermediate products at ambient temperatures. So far, systematic data on the effect of extrinsic factors on cereulide synthesis has been lacking. Thus, we investigated the influence of temperature, a central extrinsic parameter in food processing, on the regulation of cereulide synthesis on transcriptional, translational and post-translational levels over the growth temperature range of emetic *B. cereus*. Bacteria were grown in 3°C interval steps from 12 to 46°C and cereulide synthesis was followed from *ces* gene transcription to cereulide toxin production. This systematic study revealed that temperature is a cardinal parameter, which primarily impacts cereulide synthesis on post-transcriptional levels, thereby altering the composition of cereulide isoforms. Our work also highlights that the risk of cereulide production could not be predicted from growth parameters or sole cell numbers. Furthermore, for the first time we could show that the formation of the recently identified cereulide isoforms is highly temperature dependent, which may have great importance in terms of food safety and predictive microbiology. Notably the production of isocereulide A, which is about 10-fold more cytotoxic than cereulide, was specifically supported at low temperatures.

## Introduction

The incidence of foodborne intoxications caused by bacterial toxins has been steadily increasing over the last decade. Especially, the number of reported food poisonings related to *Bacillus cereus* toxins has shown a steep increase from 2006 onward (Anonymous, 2009, 2015). The rising seriousness of *B. cereus* is also reflected in the growing number of reports on severe intoxications related to the emetic *B. cereus* toxin cereulide, requiring hospitalization or even leading to death (Dierick et al., 2005; Posfay-Barbe et al., 2008; Ichikawa et al., 2010; Naranjo et al., 2011; Ehling-Schulz and Messelhäusser, 2012). Because of the high toxicity of cereulide and the high incidence rates up to 100%, usually observed in connection with outbreaks, accurate data on contamination sources and factors promoting toxin formation are urgently needed to prevent contamination and toxin production in food production processes. Especially comprehensive prevalence data on potential hazardous sources from the food processing chain are missing, and critical downstream processing steps at risk for cereulide production are hitherto unknown. Since *B. cereus* is a ubiquitous spore-former, it cannot be totally avoided in many raw material and food products. Toxin formation is of serious concern in food processing procedures that include slow cooling procedures and/or storage of intermediate products at ambient temperatures. Because of its chemical structure, the emetic depsipeptide toxin cereulide shows an extreme pH and heat stability. Once pre-formed in food ingredients or matrices, this toxin will most likely not be destroyed or inactivated throughout the food production processes. Due to its size (1.2 kDa), the toxin cannot be removed by conventional filtration and survives subsequent thermal processing (Agata et al., 1995; Rajkovic et al., 2008). For instance, contaminations with cereulide or cereulide-producing bacteria have been reported from infant foods in Finland, various food products in Belgium and Bavaria as well as from ice creams in Germany (Shaheen et al., 2006; Rajkovic et al., 2007; Messelhäusser et al., 2010, 2014).

Cereulide is a dodecadepsipeptide, composed of alternating α-amino and α-hydroxy acids (D-*O*-Leu–D-Ala–L-*O*-Val–L-Val)_3_, that is produced non-ribosomally by an enzyme complex with an unusual modular structure, named cereulide synthetase (Ces NRPS; Ehling-Schulz et al., 2005b; Magarvey et al., 2006). Very recently, it has been shown that, in parallel to the known cereulide, at least 17 different isoforms are synthesized by the Ces NRPS, which represents a novel type of a NRPS (Marxen et al., 2015a,b). The cereulide isoforms significantly differ in their cytotoxic properties. Isocereulide A shows an 8- to 10-fold higher cytotoxicity than cereulide, while some other isoforms are almost non-toxic (Marxen et al., 2015a). All emetic *B. cereus* strains so far analyzed (*n* = 80) produced all the same isoforms although at different ratios (Marxen et al., 2015a,c).

The *ces* gene locus responsible for the cereulide synthesis is encoded on a 270kb mega virulence plasmid related to the *Bacillus anthracis* toxin plasmid pXO1 (Ehling-Schulz et al., 2006; Rasko et al., 2007), and the *ces* genes have been shown to be polycistronically transcribed from a central promoter (Dommel et al., 2010). The cereulide synthesis is intrinsically controlled by a complex and tightly regulated transcriptional network that ensures correct timing of *ces* gene expression within a short period of the cell cycle (Lücking et al., 2009, 2015; Dommel et al., 2011; Frenzel et al., 2012). Although the exact mechanisms triggering cereulide production are far from being understood, latest research clearly illustrates that cereulide synthetase gene expression and cereulide toxin production is influenced by complex interactions of various intrinsic as well as extrinsic factors (for review see Ehling-Schulz et al., 2015). While significant progress has been made on the understanding of the intrinsic factors embedding cereulide synthesis in the bacterial life cycle, systematic studies on the influence of external factors on cereulide synthesis are missing. This study therefore aimed to gain new insights into the regulation of cereulide production by examining the influence of temperature, a key external parameter in food processing, on cereulide synthesis on a transcriptional, translational and post-translational level.

## Materials and Methods

### Bacterial Strains

For this study, four emetic *B. cereus* strains were used: two well-characterized strains isolated in frame of food borne outbreaks, the emetic reference strain F4810/72, isolated from vomit (also designated AH187; NCBI Reference Sequence: NC_011658) and the strain F5881, isolated from Chinese takeaway fried rice, (detail for these strains can be found in Ehling-Schulz et al., 2005a), and, further two strains from food industrial environments, one strain isolated from wheat flour (B626) and one strain isolated from dehydrated puree with onions (AC01).

### Growth Conditions and Determination of Sampling Points

Bacterial overnight cultures, pre-grown in 3 ml LB broth under laboratory standard conditions (pH 7.0, 30°C, 120 rpm), were used for kinetic inoculation (final inoculum 10^3^ cfu/ml) of the main cultures (100 ml LB broth, pH 7.0, and 120 rpm) in baffled flasks as described previously (Dommel et al., 2011). Cultures were incubated under rotary shaking at different temperatures in the range from 12 to 46°C in 3°C intervals. At least two independent growth experiments were carried out for each temperature.

**FIGURE 1:**
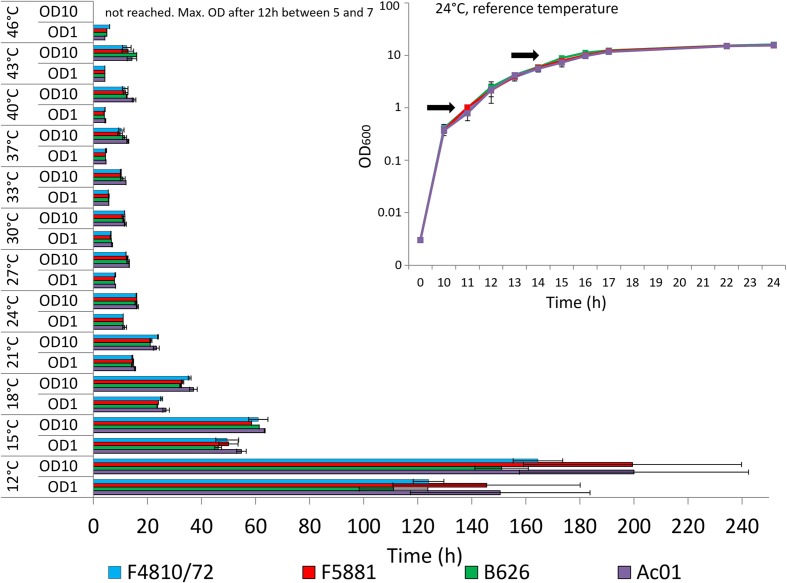
**Growth of emetic *Bacillus cereus* strains in dependence of temperature.** Strains were grown in LB broth, 120 rpm, at different temperatures in a range from 12 to 46 °C, using 3°C intervals. Time (in hours) to reach optical densities (OD_600_) of 1 and 10, at which samples have been taken in parallel for transcriptional, translational and post-translational analyses, is depicted. At 46°C, OD_600_ of 10 was not reached, and samples for transcriptional, translational and post-translational analyses were taken after 12 h when strains reached a maximal OD_600_ between 5 and 7. The inset show growth curves from all four strains recorded at 24°C, which served as reference temperature. Since all strains showed maximal *cesB* transcription and translation around an OD_600_ of 10, this OD was chosen for transcriptional, translational and cereulide toxin analysis at all tested temperatures. In addition, samples were taken at OD_600_ of 1 to be used as internal calibrator for transcriptional analysis (**Figure 2A**). Arrows indicate the OD_600_ sampling points at all tested temperatures. Data represent means and error bars indicating standard deviations of at least two independent growth experiments.

According to preliminary tests, all strains showed almost identical growth and comparable values of optical (OD_600_) and cell (cfu/ml) densities at 24°C (**Figure 1**, inset). Under these standard conditions, maximal rates of transcription and translation of *cesB* could be determined for all strains at approx. OD_600_ of 10, which is in line of results from a previous study carried out at 30°C (Frenzel et al., 2012). Thus, growth of bacteria was investigated photometrically and samples were taken at OD_600_ of 10 from bacterial cultures grown at different temperatures for further processing as outlined below. For transcriptional analyses, additional samples were taken at OD_600_ of 1.

### Transcriptional Analyses of *cesB*

Transcription of the *ces* gene *cesB* was analyzed by qRT-PCR as described previously (Dommel et al., 2011). In brief, RNA was isolated from frozen cell pellets via TRIzol Reagent (Invitrogen) and bead beating. Phase separation was carried out with chloroform and nucleic acid was precipitated with 75% ethanol. Subsequently, DNA was digested with RQ1 DNAse I (Promega) and a total amount of 1 μg RNA was used for cDNA synthesis (cDNA qScript Supermix, Quanta Biosciences). qRT-PCR for each sample was performed in triplicates on a C1000 Touch Thermal Cycler CFX96 Real-Time System (BioRad). In order to normalize and compare the relative expression the REST method was employed (Pfaffl et al., 2002), which has been shown previously to be a quite suitable tool for analysis of *ces* transcription (see, e.g., Lücking et al., 2009, 2015; Dommel et al., 2010, 2011; Frenzel et al., 2012). As internal calibrator, (with a relative expression value of 1.00) *ces* gene expression at an OD_600_ of 1.0 was chosen since it has been shown previously to be consistent throughout experiments (Dommel et al., 2011). Samples at OD_600_ of 1 and 10 were always taken from the same cultures. Sample to sample variation was corrected by using the 16S rDNA gene as a reference (rrn). Mean values and standard deviations were calculated from two independent experiments.

### Translational Analyses of CesB

Ces translation was investigated by immunoblotting using a recently generated monoclonal antibody specific for CesB (Lücking et al., 2015) and a peroxidase-conjugated secondary antibody (AffiniPure Goat Anti-Mouse IgG, Dianova). Western blot analysis was carried out according to Lücking et al. (2015). Briefly, SDS-PAGE was performed according to Laemmli (1970) and immunoblotting was performed on a BioRad Transblot SD Semi Dry Transfer Cell. Determination of total protein load was carried out by staining with Ponceau S Solution (Applichem). For the chemiluminescent reaction, Super Signal West Pico Chemiluminescent Substrate (Thermo Scientific) was applied and quantification was performed with the ImageQuant TL Software (GE Healthcare Life Sciences). At least two technical replicates per sample were performed. Relative translation of CesB was determined by calculating all CesB Western Blot signals in relation to the CesB blot signal with highest intensity (AC01, 21°C), which was set to 100%. Blot to blot variation was corrected by using CesB protein as a reference included in all blots, including at least two technical replicates per sample. Mean values and standard deviations were calculated from two independent experiments.

### Cereulide and Isocereulide Quantitation by Means of Ultra Performance Liquid Chromatography (UPLC) Tandem Mass Spectrometry (MS/MS)

For the extraction of cereulide, bacteria grown in LB medium were pelletized by centrifugation (8.000 g, 23°C, 10 min) and 50 mg of bacterial biomass was resuspended in 1 ml acetonitrile (99%, HPLC grade, Carl Roth). After incubation for 16 h on a rocking table, the pellet was centrifuged and the supernatant was directly transferred to a HPLC vial and stored at room temperature. MS measurements were performed using a QTRAP^®^ 5500 MS/MS system (Applied Biosystems, Foster City, CA, USA) equipped with a TurboV electrospray ionization (ESI) source and a 1290 series UHPLC system (Agilent Technologies, Waldbronn, Germany). Chromatographic separation was performed at 25°C on a Gemini^®^ C_18_ – column, 150 × 4.6 mm i.d., 5 μm particle size, equipped with a C_18_ security guard cartridge, 4× 3 mm i.d. (all from Phenomenex, Torrance, CA, USA). Stock solutions of 500 ppm cereulide (Chiralix, Nijmegen, The Netherlands) and 100 ppm ^13^C_6_- cereulide (Chiralix) were prepared for the standard curve and internal standard, respectively. MS-analysis was performed as described by Bauer et al. (2010) and quantitation of isocereulides was carried out as reported previously (Marxen et al., 2015c). All samples were measured in two different dilutions as duplicates. Mean values and standard deviations were calculated from two independent experiments.

### Protease Activity Measurement

Determination of protease activity was carried out with Pierce Fluorescent Protease Assay kit (Thermo Scientific), according to manufacturer’s instructions and spectrophotometric measurement was carried out as described above.

### Statistical Analysis

Data were analyzed with R (version 3.3.1) software (Hornik, 2016) using the Welch’s *t*-test to evaluate the null hypothesis that temperature does not affect the tested parameter. Data from experiments carried out at 24°C served as reference for each strain. A *P*-value of 0.01 or less was considered statistically significant. Data represent means and error bars showing standard deviations from at least two independent experiments.

## Results

The influence of temperature on cereulide synthesis of *B. cereus* grown in LB medium was investigated photometrically at a wavelength of 600 nm (OD_600_). Preliminary kinetic analyses carried out at the reference temperature 24°C, revealed a maximum of *cesB* transcription and CesB translation around OD_600_ of 10 for all four strains included in this study (data not shown). Thus, OD_600_ of 10 was chosen as sampling point. To cover the full growth range of emetic strains, bacterial cultures were grown from 12°C to 43°C in 3°C intervals to OD_600_ of 10 and at 46°C, a temperature at which OD_600_ of 10 was not reached, up to the maximum OD_600_). From cultures of all four strains grown at the different temperatures to an OD_600_ of 10 (or to the maximum OD_600_ at 46°C), samples were taken in parallel for transcriptional, translational and post-translational analysis of cereulide synthesis. Samples were processed as described previously (Frenzel et al., 2012; for details see also material and method section). For transcriptional analysis, additional samples were taken from all cultures at OD_600_ of 1 to be used as internal calibrator (Dommel et al., 2011).

### Effect of Temperature on Growth of Emetic *B. cereus*

An overview on the time needed to reach OD_600_ of 1 and OD_600_ of 10, respectively, is provided in **Figure 1**. The inset shows the growth curves for all strains at the reference temperature of 24°C. The fastest start of growth was observed at temperatures >33°C, reaching an OD_600_ of 1 within 4–4.5 h (37°C: 4.5 h, 40°C: 4.0 h, 43°C: 4.25 h, and 46°C: 4.25 h). However, at temperatures exceeding 37°C, the time needed to reach OD_600_ of 10 was slightly longer, compared to the time needed at 37°C. All strains reached OD_600_ of 10 between 33 and 37°C after 10–12 h, while at 46°C none of the strains reached OD_600_ of 10. At the latter temperature, a maximal OD_600_ of 5–7 was observed for all strains after 12 h, thereafter the OD_600_ values dropped (data not shown), pointing toward severe growth temperature stress around the upper growth temperature boundary. Generally, below 18°C, all strains showed slow growth and the cell division rate was significantly reduced. Around the lower growth temperature boundary at 12°C, OD_600_ of 1 was reached after 4.5–6 days and the time needed to reach OD_600_ of 10 required 7–9 days, respectively. At room temperature (24°C), all strains showed the same growth behavior while at the growth boundaries some strain-specific growth behavior was observed. Strain AC01 tends to be the slowest growing strain, especially at low temperatures. In contrast, strain B626 was growing faster at low temperatures than the other strains while it appeared to be the slowest growing strain at temperatures >40°C, raising the question whether this strain is more adapted to low temperatures than the other emetic strains included in this study.

### Effect of Temperature on *cesB* Transcription and Translation

**FIGURE 2:**
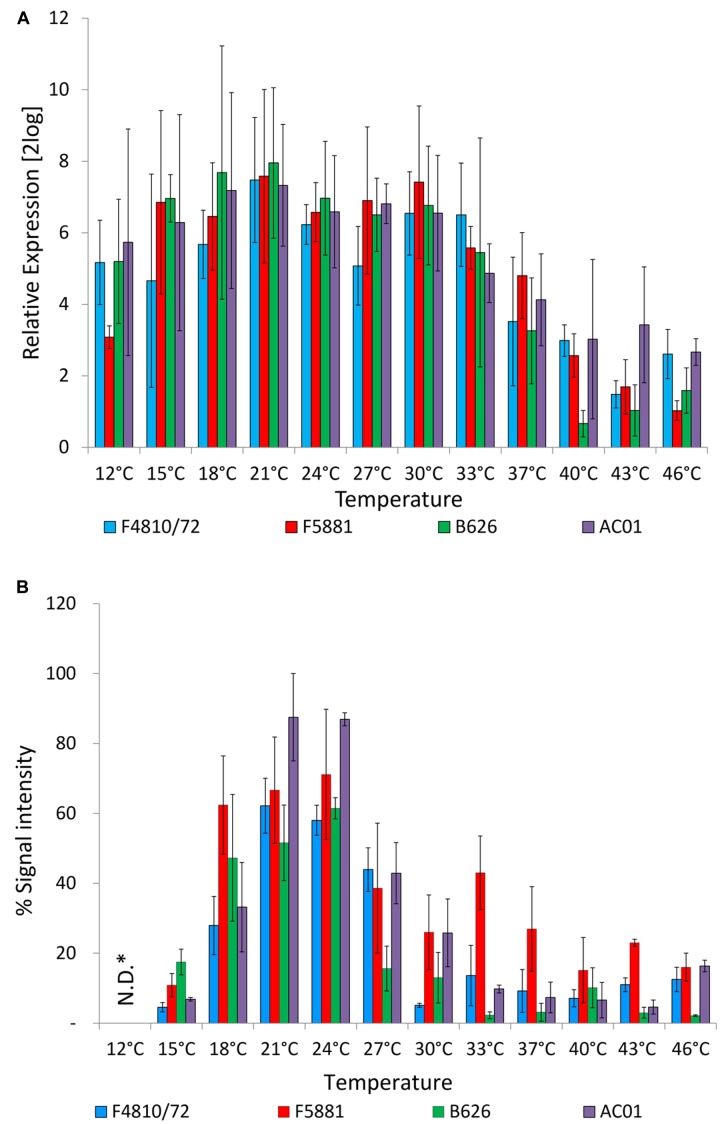
**Cereulide synthetase expression in dependence of temperature.** Cultures were grown to an OD_600_ of 10 in LB broth, 120 rpm, at different temperatures in a range from 12 to 46 °C, using 3°C intervals. Samples were taken for *cesB* gene transcription **(A)** and CesB translation analysis **(B)**. Transcription was analyzed by qRT-PCR according to Dommel et al. (2011) and translation was analyzed by immunoblotting using a novel CesB-specific monoclonal antibody (Lücking et al., 2015). For relative expression analyses of *cesB* at OD_600_ of 10, the *cesB* transcript level at an OD_600_ of 1 was used as the calibrator (RE = 1.0) for each strain at all tested temperatures. Relative translation of the CesB protein at different temperatures was determined by calculation CesB blot signal intensity of each sample relative to CesB showing highest blot signal intensity (RE = 100%). Data represent means and error bars indicate standard deviations of at least two independent growth experiments. ^∗^N.D., not determined.

The influence of growth temperature on transcription and translation of the cereulide synthetase was determined by quantitative RT-PCR detecting *cesB* mRNA levels and immunoblotting, using a CesB specific monoclonal antibody (Lücking et al., 2015). The *ces* genes *cesP,T,A,B,C,D* are transcribed polycistronically from a main promoter upstream of *cesP* and qRT-PCR revealed the same transcriptional kinetics for *cesP,T,A,B,C,D* (Dommel et al., 2010, 2011; Frenzel et al., 2012). Currently, only for CesB a specific antibody (CesB mAB) is available but not for any of the other Ces proteins encoded by the *ces* operon. Thus *cesB*/CesB was chosen for our survey to use the same target on transcriptional and translational level. Unexpectedly, no significant differences on transcript levels of *cesB* (*P* < 0.01) were observed over a broad growth temperature range from 12°C or 15–33°C, depending on the strain. A decrease of the temperature even to the lower growth boundary (12°C) did not reveal a significant difference in *cesB* transcription in strain AC01 compared to the *cesB* transcription level at 24°C, which served as reference temperature, while a significant decrease of *cesB* transcription was found for the other strains at 12°C (*P* < 0.01). An increase of the temperature from 24 to 37°C resulted in significant decrease of *cesB* transcription in all four strains (*P* < 0.01). At higher temperatures, transcription was declining but still detectable up to the upper growth boundary (46°C; **Figure 2A**). Ces translation was also observed over the complete growth range. However, in contrast to *cesB* transcription, translation turned out to be highly temperature dependent (**Figure 2B**). A steep increase of the CesB protein signal in the immunoassay was observed from 15 to 18°C. Between 21 and 24°C the plateau of maximal CesB translation was reached in all strains. No significant differences were found in CesB levels between 21 and 24°C (P < 0.01). At higher temperatures, the CesB signal was dropping in a strain-specific manner. For instance, the signal in the CesB-specific immune assay for AC01 and B626 at 27°C dropped to approximately 50 and 25%, respectively, of the signals detected at 24°C. The CesB signal for the emetic reference strain F4810/72 sharply declined at 30°C, while the decline of the CesB signal of the strain F5881 at high temperatures was not as pronounced as for the other strains. Remarkably, strain B626, which appeared to be the best adapted strain to low temperatures among the strains used in this study, showed the lowest CesB levels in most cases, except at 15°C, at which the highest signal of all strains was observed. CesB protein signals were also detectable for all strains at 12°C, but could not be quantified due to the severe growth impairments and cell damages at the lower growth boundary (**Figure 1**).

Additionally, a protease assay was employed to test if the drastic drop of CesB signals from 18 to 15°C is caused by increased protease activity at the lower temperature. However, results from a comparative study of protease activity from bacterial cultures grown at 24 and 15°C to OD_600_ of 10 and accompanying CesB stability test did not reveal a decreased stability or specific degradation of CesB at 15°C (data not shown). Thus, it is more likely that the differences observed at 18 and 15°C are the result of temperature dependent CesB translation rather than the consequence of CesB degradation.

### Effect of Temperature on Cereulide Toxin Production

**FIGURE 3:**
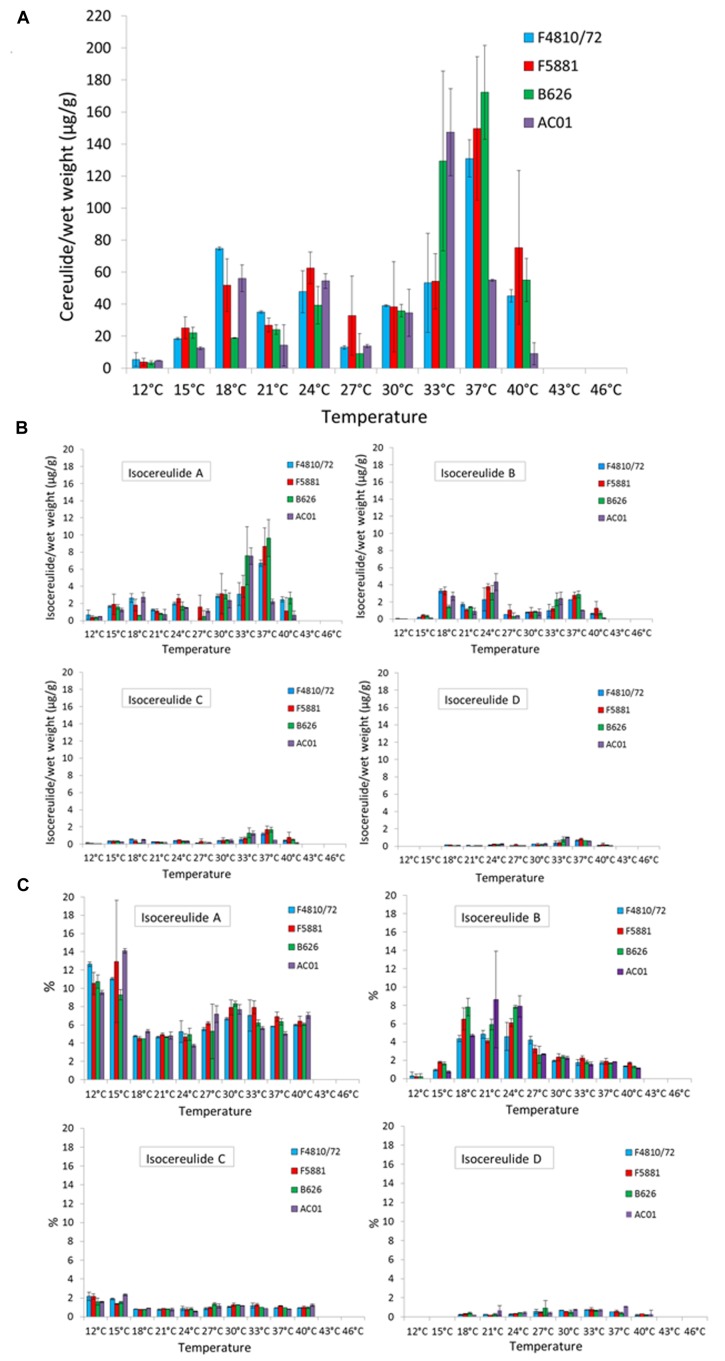
**Cereulide toxin and isocereulide production in dependence of temperature.** Cultures were grown to an OD_600_ of 10 in LB broth, 120 rpm, at different temperatures in a range from 12 to 46 °C, using 3°C intervals. Cereulide and isocereulide extraction was carried out with acetonitrile (99%) and quantitation was performed by ESI-HPLC MS/MS analysis. Cereulide **(A)** and isocereulide **(B)** levels are depicted as μg cereulide per g bacterial wet weight as well as relative amount of cereulide isoforms, referred to the amount of cereulide values, are shown **(C)**. Data represent means and error bars indicating standard deviations of at least two independent growth experiments.

Whereas the *cesB* transcript and CesB protein were detectable over the whole temperature range, the toxin was not detectable at the upper growth boundary (46 and 43°C) and only at very low levels at the lower growth boundary (12°C; **Figure 3A**). For instance, at 12°C toxin amounts between 2 and 8 μg/g bacterial wet weight were found in the analyzed strains, while the highest toxin amounts—ranging from 31–194 μg/g bacterial wet weight—were detected at 33 and 37°C, respectively. Paralleling the results from the translation analyses, a steep increase in cereulide toxin amounts was observed when the growth temperature was raised from 15 to 18°C. A second and even more pronounced increase in cereulide amounts was found when temperature was shifted from either 30 to 33°C (AC01 and B626) or from 33 to 37°C (F4810/72 and F5881), indicating a strain-specific response to growth temperature. For instance, at 33°C strain F4810/72 produced about 50 μg/g and AC01 produced about 130 μg/g, whereat the cereulide levels detected at 37°C were completely reverse in these two strains (**Figure 3A**). A maximum of cereulide toxin was detected for one out of the four strains at 33°C (AC01: 147 μg/g bacterial wet weight) and for the other three strains at 37°C (F4810/72: 131 μg/g bacterial wet weight, F5881: 149 μg/g bacterial wet weight, B626: 172 μg/g bacterial wet weight). After the maximum of toxin accumulation was reached, further increase of the temperature to 40°C caused a sharp decline in toxin amounts in all strains tested, down to levels comparable to those between 18 and 24°C. A further increase of the temperature to 43°C resulted in an almost complete stop of cereulide production, although all strains showed fast growth. The maximum toxin levels at 43°C ranged from 0.003 to 0.09 μg/g wet weight. Thus, the risk of cereulide toxin formation cannot be predicted from sole cell numbers or growth parameters. Interestingly, cereulide levels found at 40°C were similar to that of 18–30°C, but *cesB* transcription and CesB expression were significantly lower at 40°C.

### Effect of Temperature on Isocereulide Production

Recently, 17 isoforms of cereulide—apart from the known cereulide—have been discovered and a novel method for multiparametric quantitation of isocereulides A–G was developed (Marxen et al., 2015a,c), which provided us the possibility to examine the influence of temperature on the composition of isocereulides. Our study showed that isocereulide A and isocereulide B were produced in LB-grown bacterial cultures up to levels of 14% of the ones observed for cereulide, while isocereulide C and D were produced at levels equaling 1–2% of cereulide (**Figures 3B,C**). Most interestingly, highest amounts (9–14%) of the highly cytotoxic isocereulide A were found at low temperatures (12 and 15°C), while relative low levels of isocereulide B (0.8–1.8%) were detected at these temperatures. However, the latter isoform was produced to levels up to 10% between 18 and 27°C, corresponding to the temperature range in which comparable low amounts of isocereulide A were detected. Isoforms C and D were below 2%, whereas isoform D was not detectable at low temperatures (12 or 15°C). Therefore, it is tempting to speculate that emetic strains are shifting the production of isocereulide A and isocereulide B in a temperature dependent manner, which must be taken into account during risk assessments in the food industry.

## Discussion

### Temperature: A Cardinal Environmental Effector of Post-transcriptional Cereulide Synthesis Regulation

Cereulide synthesis in emetic *B. cereus* is controlled by a network of bacterial intrinsic factors, involving different realms of regulation (for review see Ehling-Schulz et al., 2015). These bacterial intrinsic factors include key factors, which act on the transcriptional level (Lücking et al., 2009; Frenzel et al., 2012), thereby embedding cereulide production in the bacterial lifecycle (Dommel et al., 2011). Transcription of *ces* genes is highly dynamic and restricted to a distinct growth phase (Dommel et al., 2011; Frenzel et al., 2012; this work). Very recently it has been shown that in addition to these chromosomally encoded central transcription factors, proteins embedded in the cereulide synthetase (*ces*) locus exert control of the non-ribosomal synthesis of cereulide on transcriptional, translational and post-translational levels (Lücking et al., 2015). We therefore aimed in the current work to assess the influence of temperature as a key extrinsic factor in food processing environments on the different levels of cereulide synthesis control. In contrast to intrinsic factors, which are tightly regulating cereulide synthesis on a transcriptional level, temperature—as an extrinsic signal—influenced toxin expression mainly on a post-transcriptional level. No significant differences on transcript levels of *cesB* were observed over a broad growth temperature range (**Figure 2A**). At higher temperatures, transcription was declining but still detectable up to the upper growth boundary. In comparison, studies on the effect of temperature on the botulinum toxin complex L-TC have shown higher mRNA levels at lower temperatures and lower transcript levels as well as less stability at high temperatures (Couesnon et al., 2006; Chen et al., 2008). Therefore, considering the differential regulation of CesB protein and cereulide, it is tempting to speculate that temperature sensing of the cell triggers a response of cereulide synthesis on a translational and/or post-translational level rather than on a transcriptional level.

It was also noticeable that at the upper growth boundary the CesB protein is still formed but apparently not the toxin. Diminished cereulide levels at 21 and 24°C are not in coherence to the high expression of CesB at these temperatures. Thus, it can be assumed that temperature has rather a post-translational regulatory effect, and may orchestrate the production of the toxin out of the dipeptides by the cereulide synthetase—but not the expression of the single NRPS modules—or may influence the activity of Ces NRPS in another, hitherto unknown way. Very recently, a novel mechanism for the action of cereulide synthetase has been proposed, highlighting dipeptides rather than single amino or hydroxy acids as the basic modules in toxin assembly (Marxen et al., 2015b). Further studies are needed to fully understand the mechanism of cereulide toxin synthesis and export as well as the exact role of external factors, such as temperature, on this highly complex process.

### Temperature: A Key Parameter for Cereulide Production

Although temperature abuse is one of the key factors in emetic food poisoning caused by *B. cereus* (Ehling-Schulz et al., 2004; Dierick et al., 2005; Ehling-Schulz and Messelhäusser, 2012), information on the influence of temperature on cereulide synthesis in emetic *B. cereus* is still limited. Generally, emetic strains show a shift of their growth temperature limits toward higher temperatures compared to non-emetic strains (Carlin et al., 2006). These results are in line with those from our current study (**Figure 1**). Temperatures below 21°C led to strongly decelerated growth, with long pre-exponential and exponential phases. Generally, the time needed to reach OD_600_ of 1 was decreasing with increase of growth at temperatures up to 43°C, pinpointing the temperature shift of emetic strains toward higher temperatures compared to non-emetic *B. cereus* strains. All strains reached OD_600_ of 1 at 40 and 43°C after 4 h. Around the temperature growth boundaries, a 3°C change significantly impacted the growth behavior of all strains, although, in a different way at the lower than at the upper temperature growth boundary. At 12°C, growth was generally retarded, while at 46°C the strains reached a maximum OD_600_ of 5–7 already after 12 h, thereafter the OD_600_ values were dropping (data not shown). Thus, it is tempting to speculate that bacteria successfully start growing at high temperatures but are rapidly accumulating cell damages caused by heat stress (e.g. radical formation, malfunction of chaperons, etc.). In contrast, at the lower growth boundary OD_600_ of 10 was reached after seven to 9 days, which may rather reflect general growth impairments than acute cell damages. The latter must be taken in consideration when interpreting results of cereulide expression studies.

Various studies have analyzed the production of cereulide under different temperature conditions in laboratory broth, on agar plates or in different food matrices (e.g., Finlay et al., 2000; Häggblom et al., 2002; Rajkovic et al., 2006; Shaheen et al., 2006; for review see Ehling-Schulz et al., 2004). However, results are difficult to compare due to the lack of standardization for cultivation (pre-culture conditions, inoculation levels, and media) and methods used for cereulide quantitation (bioassays, cytotoxicity assays, and mass spectrometry). For instance, it has been shown that history of a strain could affect strain capacity for cereulide production (Thorsen et al., 2009; Dommel et al., 2011). Furthermore, to our knowledge, hitherto no systematic analysis on the effect of temperature on cereulide production in emetic strains has been performed. Thus, we systematically investigated cereulide production over the complete growth temperature range using narrow temperature intervals. As shown by this approach, an increase of only 3°C at certain temperatures resulted in a twofold (e.g., for F4810/72: from 5 μg/g at 12°C to 11 μg/g at 15°C) or even more than fivefold (e.g., for F4810/72: from 7.5 μg/g at 27°C to 43.9 μg/g at 30°C) increase of cereulide levels, while a further increase of 3°C, after the maximum of cereulide production was reached, resulted in a drastic drop of cereulide levels (**Figure 3A**), pinpointing the importance of systematic and in-depth analyses, such as carried out in the present work. Corroborating results from previous studies, we found detectable, although low cereulide levels at the lower growth temperature boundary. Generally, 12°C seems to be the minimum temperature limit for cereulide production (for review see Ehling-Schulz et al., 2004). Finlay et al. (2000), who studied cereulide production in skim milk medium at various temperatures, detected toxin in a temperature range between 12 (after 4 days) and 37°C (after 24 h), which is in principle in line with our results. However, in contrast to the findings of Finlay et al. (2000), we found the highest levels of cereulide at 33–37°C and not at low temperatures. Probably these discrepancies might be explained by the different approaches used for quantitation of cereulide. Finlay et al. (2000) used a cell culture assay for cereulide quantitation, which cannot discriminate the different isocereulides, while in the current study mass spectrometry based systems for accurate quantitation of cereulide and isocereulides, were employed (Bauer et al., 2010; Marxen et al., 2015c).

### Temperature: The First Extrinsic Parameter Shown to Impact Isocereulide Composition

The recently discovered isocereulides, which are characterized by alternating positions of the dipeptides within the cyclic dodecadepsipeptide ring structure, significantly differ in their cytotoxic properties (Marxen et al., 2015a,b). For instance, isocereulide A shows an 8- to 10-fold higher cytotoxicity than the classical cereulide while isocereulide B is almost non-toxic (Marxen et al., 2015a). If or how external parameters, such as temperature, are influencing the composition of isocereulides, was hitherto unknown. We therefore aimed in this study to assess the effect of temperature on the occurrence and quantity of cereulide isoforms, using a novel method for simultaneous detection and quantitation of isocereulide A–G (Marxen et al., 2015c). To our knowledge, this is the first study investigating the influence of an extrinsic factor on the production of isocereulides. The isocereulide levels found in our study were in the same %-range as those from samples of recent foodborne outbreaks (Marxen et al., 2015c). Isocereulide A was detected in all samples tested positive for cereulide (**Figure 3**). Our results show a dramatic shift of the relative amount of isocereulide A and isocereulide B at the boundary of room temperature, at 18°C, a temperature that also represents a threshold for significantly slowed growth. Thus, low temperatures turned out to specifically support the production of this highly toxic isoform. This finding makes low temperatures a new critical parameter in terms of food hygiene and storage, although it must be kept in mind that the absolute amount of isocereulide A is still higher at 33 and 37°C, due to generally elevated cereulide levels at the latter temperatures.

## Conclusion

Our systematic investigation of temperature, a cardinal parameter in cereulide toxin synthesis and critical factor in food processing environments, showed that temperature exerts control of cereulide synthesis in emetic *B. cereus* primarily on post-translational levels. How these external signals are embedded in the bacterial lifecycle and internal signaling is largely unknown and warrants further investigation. The cereulide toxin production ceased at temperatures at which fastest growth was observed, highlighting that the risk of toxin formation cannot be deduced from sole cell numbers or growth parameters. To our knowledge, this study is the first one investigating the effect of temperature on the formation of isocereulides. Importantly, low temperatures turned out to specifically support the production of the highly toxic isocereulide A. The amounts of isocereulides found in our study paralleled those from recent food-borne outbreaks. Thus, further studies are needed to systematically investigate the influence of environmental factors on the production of isocereulides and to assess the consequences of these novel, unexpected findings for *B. cereus*-related risk assessments.

## Author Contributions

Performed the experiments: MK, KS, and MS; Analyzed the data: MK, KS, MS, and ME-S. Conceived and designed the experiments: ME-S, KR-S, and LB. Wrote and revised the paper: MK, ME-S, KR-S, and LB; ME-S has conceptualized, supervised, and acted as overall study director.

## Conflict of Interest Statement

The authors declare that the research was conducted in the absence of any commercial or financial relationships that could be construed as a potential conflict of interest.
